# Event and Comment

**Published:** 1919-03

**Authors:** 


### DENTAL REGISTER

THE MAGAZINE OF DENTISTRY

Vol. LXXIII | MARCH, 1919 | No. 3

### EVENT AND COMMENT

**The Next Move in Dental Education** It may seem rather premature to contemplate a new advance in the educational standards in view of the fact that most colleges have scarcely got the four year curriculum well started. It is announced by the New York Board of Regents that it will require specific preparation for students entering the dental course, in the way of unconditional complete high school graduation credentials in which certain subjects are mandatory; such as English, Physics, Biology and Chemistry. It may be fair for people outside of the state of New York to question the propriety of such a requirement in view of the fact that other states may not see fit to make a similar requirement. But, what will they say when it becomes known that students graduating from colleges which do not require such preliminary training before being admitted to the dental course, will not be permitted to register for practice in New York state, for the reason that they have not graduated from colleges that are fully registered with the New York Board of Regents? When the significance of this action is realized we shall see that New York in this matter has made a most radical advancement of its professional requirements. In a few words it means that all colleges that will not make this requirement a prerequisite for matriculation will find New York state closed to their graduates. It may be interesting in this connection to state that only about half of our best high schools teach these subjects fully.

New York has undoubtedly taken this step advisedly, and while it would seem at first thought to be a somewhat provincial and selfish motive that inspires such an action at this time, after all, its effect will be good for educational standards as it will establish an official standard of preliminary education for admission to dental colleges in one state at least, and certainly for all colleges outside of that state that receive New York students. New York is also leading a movement to require in 1921 a year of pre-dental work in a standard literary college as a requirement for admission to its dental colleges. This advancement is also being contemplated in other states and may be the next move in advancing educational standards, although it is more than probable that the larger number of dental colleges in other states would not be equal to such an advance. It is possible that most of the universities and endowed colleges could carry out such a program, but it would seriously tax the resources of the privately owned smaller schools, and many of them would undoubtedly go out of existence. It is interesting to remember in this connection that New York state where this movement originates has no university or college that is supported, except by private means. What the effect of such legislation on these colleges will be it is difficult to foresee. We suppose they must be cooperating with those in authority and know what to expect from such legislation. Under present conditions the attendance in dental colleges is surely decreasing and the public demand for dental service is rapidly increasing, and it is difficult to foretell what the result may be. It is almost certain that we are to see in the near future some unexpected developments in our profession, consequent of the prevalent tendency to overthrow the present order and enthrone the idealist. It would seem that it would be much better if matters that mean so much to our profession should receive ample and general consideration before such far reaching steps are inaugurated by any important body.

